# Obesity Triggers Dysregulation of Essential ABC Transporters in Rat Testis and Sperm

**DOI:** 10.3390/nu18111829

**Published:** 2026-06-05

**Authors:** Péter Szatmári, Kata Kira Kemény, Adrienn Seres-Bokor, Eszter Ducza

**Affiliations:** Department of Pharmacodynamics and Biopharmacy, Faculty of Pharmacy, University of Szeged, Eötvös Street 6, 6720 Szeged, Hungary; szapeti40@gmail.com (P.S.); kemeny.kata.kira@szte.hu (K.K.K.); seres-bokor.adrienn@szte.hu (A.S.-B.)

**Keywords:** obesity, testis, sperm, P-glycoprotein, breast cancer resistance protein, rat, ABC transporter

## Abstract

**Objectives:** Obesity and the associated metabolic dysfunction influence fertility performance at molecular levels and ABC transporters are considered as potential molecular factors affecting fertility both in the testis and sperm; therefore, we aimed to examine the effect of a short-term diet-induced obesity on testicular and spermatic ABC transporters in a rat model focusing on the expressions of P-glycoprotein (P-gp, Abcb1) and breast cancer resistance protein (BCRP, Abcg2). The testicular androgen state involving aromatase enzyme (*Cyp19a1*), androgen receptor (*Ar*), and testosterone levels were also evaluated. **Methods:** Obesity was induced in male Sprague Dawley rats by feeding a high-fat, high-sugar diet (HFHSD) for 10 weeks, and metabolic status was evaluated using a glucose tolerance test. The weight and size of reproductive organs were measured, and Abcb1a/1b, Abcg2, *Cyp19a1*, and *Ar* expression in testes or sperm was determined by RT-PCR and Western blotting. At the same time, testosterone levels were measured by ELISA. **Results:** HFHSD successfully induced higher weight gain with glucose intolerance and reduced reproductive organ size. In obese rats, testicular *Abcb1a* and *Abcb1b* mRNA and P-gp protein expression were significantly higher, whereas testicular *Abcg2* mRNA levels decreased. Spermatic *Abcb1a*, *Abcb1b* and *Abcg2* mRNA expression also reduced in obesity. Neither testicular testosterone concentration nor *Cyp19a1* and *Ar* mRNA expression levels changed after the 10-week obesogenic diet compared with controls. **Conclusions:** Overall, our study revealed infertility-related ABC transporter changes in obese male rats, suggesting that these alterations may predispose obese males to fertility impairments, even before the obesity-induced androgen dysregulation.

## 1. Introduction

Obesity has been rapidly widespread in recent decades and is expanding continuously worldwide, holding the leading position of metabolic disorders among the adult population. The latest reports estimate that more than 1 billion people are obese globally, which could increase by one and a half times within the next ten years [1]. The extended adipose tissue becomes a significant metabolic and endocrine organ by secreting bioactive adipocytokines, enzymes, and receptors involved in steroid metabolism and response [2,3]. Adipose tissue is an important site of sexual steroid metabolism, as it expresses steroidogenic enzymes, including cytochrome P450-dependent aromatase (CYP19A1), which converts peripheral androgens to estrogens [2]. Massive expansion of adipose tissue mediates CYP19A1 expression, contributing to lower serum testosterone and elevated estrogen levels. The shifted androgen-estrogen ratio in the plasma can disrupts the hypothalamic–pituitary–gonadal axis, leading to testicular dysfunction in obese males [2,4,5]. Increased adiposity is negatively correlated with sperm quality and approximately every 10 kg of excess weight reduces the fertility rates by 10% [6]; nevertheless, the effect of obesity on sperm parameters (sperm concentration, motility, morphology, and viability) has a complex and multifactorial etiology [7,8,9] which involves several physical and molecular factors as scrotal temperature, hormonal imbalance, insulin resistance, oxidative stress or inflammation [10].

Seminiferous tubules are the functional unit of the testes and the place of spermatogenesis, which are surrounded by the blood–testis barrier (BTB, also called the Sertoli barrier). BTB is formed by Sertoli cells, with tight junctions that isolate developing sperm from the blood and provide a unique microenvironment for them [11]. Testicular ATP-binding cassette (ABC) transporters are efflux pumps that regulate the transmembrane substrate transport in Sertoli cells by binding ATP intracellularly to generate energy for a conformational switch that enables transmembrane transport against the concentration gradient [12,13]. One of the most abundant ABC transporters expressed in the testicular tissue are the P-glycoprotein (ABCB1, P-gp) and breast cancer resistance protein (ABCG2, BCRP). Their main function at the BTB is to prevent the accumulation of harmful xenobiotics in the testes, especially environmental pollutants which have significant impact on male fertility [14,15]. In addition to regulating the exposure to xenobiotics, including medicinal drugs, environmental toxicants, or dietary constituents [16], they also balance the concentrations of physiological substances within the seminiferous ducts, including androgens [17,18], steroid conjugates [14], cholesterol [19], bile acids, or uric acid [20,21]. Since paracellular and transcellular transport processes are limited, BTB permeability to chemical, biological, and pharmacological agents is essential to maintain the physiological milieu for spermatogenesis; therefore, the importance of testicular P-gp and BCRP transporter expression and function in male fertility is established [11,22].

Several studies reported that testicular and spermatic ABC transporter dysregulations are associated with fertility impairments through the altered endogenous hormonal balance of the testis or the disrupted protective function [14]. Although *Abcb1a/1b* and *Abcg2* knock-out mice are fertile, they show increased sensitivity of their substrates such as ivermectin [23], vinblastine [23], regorafenib [24], or triptolide [25], which have reproductive toxicity effects on male fertility. Reproductive parameters, including testicular, epididymal and accessory sex organ weights, as well as sperm count and motility decreased while chromosomal aberrations were increased when rats were treated with a P-gp inhibitor, verapamil, prior to ivermectin administration [26]. Moreover, males with a subfertile phenotype show increased expression of P-gp compared with normozoospermic men [27]. Spermatic P-gp is localized to the midpiece of spermatozoa and mainly defends the vulnerable spermatozoa against toxic compounds. At the same time, BCRP is primarily expressed at the acrosome region in mature sperm and is associated with the cholesterol efflux to provide higher membrane fluidity, which is critical for the initiation of the acrosome reaction and supports the hyperactivated motility for penetrating the egg [28,29,30,31]. P-gp also transports endogenous sterols, which may also play a role in the regulation of sperm capacitation and acrosome reaction [29]. Studies also revealed that genetic polymorphisms of *ABCB1* (rs1045642) and *ABCG2* (rs2231142 and rs2231137) in the semen samples are associated with male infertility [21,32].

Based on the above-mentioned existing data, the expression or function of testicular and spermatic P-gp and BCRP transporters has a great impact on fertilization ability; these ABC transporters are considered as potential molecular factors affecting fertility both in the testis and sperm. More studies revealed that obesity is able to alter the expression of ABC transporters in tissues like the intestine [33], liver [34,35], kidney [35], or placenta [16], contributing to toxicological consequences or organ dysfunctions. Since the influence of obesity on testicular and spermatic ABC transporters is currently unknown, we aimed to examine the effect of obesity on reproductive organs in a short-term diet-induced obese rat model, focusing on the expression changes in P-gp and BCRP efflux transporters in rat testes and sperm. Testosterone concentration was also measured in the testis, as it is an endogenous substrate of P-gp [17,18] and can modulate the expression of ABCG2 [36]. To reveal a detailed testicular androgen status, testosterone-related proteins, including aromatase enzyme (*Cyp19a1*) and androgen-receptor (*Ar*) mRNA expressions, were also evaluated in this research.

## 2. Materials and Methods

### 2.1. Experimental Animals

The study was approved by the National Scientific Ethical Committee on Animal Experimentation (approval number: IV/911/2025) and conducted in accordance with the ARRIVE guidelines. All animal experiments were carried out following the European Communities Council Directive (2010/63/EU) and the Hungarian Act for the Protection of Animals in Research (Article 32 of Act XXVIII).

Sprague Dawley rats were procured from INNOVO Ltd. (Gödöllő, Hungary) and maintained in a regulated environment at 22 ± 3 °C, 30–70% relative humidity, and a 12–12 h light/dark cycle.

### 2.2. Dietary Intervention

**Figure 1:**
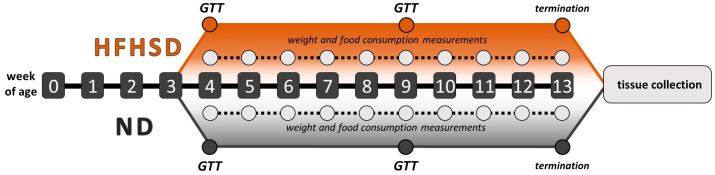
The experimental design of the 10-week dietary intervention. ○: planned interventions, GTT: glucose tolerance test, HFHSD: high-fat, high-sugar diet, ND: normal diet.

At 3 weeks of age, immature male rats (n = 20) were weaned, randomly assigned to two nutritional groups, and maintained on different diets with ad libitum access to bottled tap water. The dietary intervention is illustrated in Figure 1. Obesity was induced in rats by feeding a high-fat, high-sugar diet (HFHSD; 28% fat, 16% protein, 56% carbohydrates; 3902 kcal/kg; C1011, Altromin Spezialfutter GmbH & Co. KG, Lage, Germany). At the same time, control animals were maintained on a normal diet (ND; 14% fat, 27% protein, 59% carbohydrates; 3339 kcal/kg; C1314, Altromin Spezialfutter GmbH & Co. KG, Lage, Germany) until the end of the experiment [16]. Throughout the study, animal weights were measured weekly, and metabolic status was evaluated using a glucose tolerance test at 4 and 9 weeks of age. Food consumption was also measured weekly, and caloric intake was calculated for each animal. At the age of 13 weeks—10 weeks after the start of diets—mature rats were sacrificed by exsanguination through cardiac puncture under deep isoflurane (AErane liquid for inhalation, Baxter Hungary Ltd., Budapest, Hungary) anesthesia using a Vaportec Isoflurane Vaporiser (Burtons Medical Equipment Ltd., Kent, UK) with a Fluovac Anesthetizing System (Harvard apparatus, Holliston, MA, USA).

### 2.3. Glucose Tolerance Test

The glucose tolerance test was performed on all animals at 9 weeks of age, 6 weeks after the start of their diets. Before the experiment, rats were deprived of chow for 16 h. Blood glucose levels were measured using a glucose biosensor (Dcont^®^ ETALON ^®^ Glucose Meter, 77 Elektronika Ltd., Budapest, Hungary) in accordance with our previous studies [16,37,38].

### 2.4. Tissue Collection

Following scrotal incision, both testes were surgically removed. The penis was isolated and excised at the level of the ischial arch without the foreskin and shaft skin. A digital analytical balance was used to measure the wet weights of the tissues. The length and width of the testes and the stretched length of the penis from the tip of the glans penis to the end were measured by a metric ruler [39]. The size of the testes was estimated by calculation using the prorate spheroid formula [40]:

$$testissize={width}^{2}\times length\times 0.523$$

After the measurements, testicular tissues were frozen immediately in liquid nitrogen and stored at −80 °C until molecular analysis.

### 2.5. Sperm Isolation

The epididymis, free of associated tissue, was obtained from each rat, and the cauda was excised from the epididymis with sterile surgical scissors. Each cauda was placed in an Eppendorf tube containing 1.5 mL of pre-warmed PBS at 37 °C (ROTI^®^Fair PBS 7.4, 200 mL/tablet, Carl Roth, Karlsruhe, Germany) and was incised several times. The samples were incubated for 30 min at 37 °C (Bio-TBD 100 Dry Block Heating Thermostat, Biosan, Latvia) to release sperm. The sperm suspension was centrifuged at 4 °C for 5 min at 1000× *g* (SIGMA 1–15 K Centrifuge, Sigma Laborzentrifugen GmbH, Osterode am Harz, Germany). The sperm pellet was resuspended in 1.5 mL PBS, and aliquots were frozen at −80 °C until further experiments.

### 2.6. Sample Preparation

#### 2.6.1. RNA Isolation

From both testicular tissues, approximately 50–50 mg (from left and right testicles) slices were cut and powdered together with a ball mill (Sartorius MikroDismembrator U, Göttingen, Germany) at 2000 rpm for 1 min using liquid nitrogen. A total of 100 mg of testicular tissue or 300 µL aliquot of sperm suspension (150–150 µL from each cauda) were homogenized with 1 mL TRItidy GTM reagent (AppliChem GmbH, Darmstadt, Germany) according to the procedure of Chomczynski and Sacchi [41]. After precipitation with 500 μL of isopropanol, the precipitated RNA pellet was washed using 1 mL of alcohol and resuspended in 50 μL of AccuGene^®^ molecular biology water (Lonza, Verviers, Belgium). Concentration of RNA solutions was determined by BioSpec Nano (Shimadzu, Japan) spectrophotometer at 280 nm.

#### 2.6.2. Protein Isolation

Similarly, for RNA isolation, 100 mg of testicular tissue powder was homogenized with a 500 μL mixture of RIPA Lysis Buffer and Protease Inhibitor (Thermo Fisher Scientific, Budapest, Hungary) then shaken for 20 min at 4 °C and 900 rpm (Thermo-Shaker TS-100C, Biosan, Riga, Latvia) and centrifuged for 30 min at 11,000 rpm at 4 °C. The supernatant was pipetted into a new tube, and the protein concentration of the samples was measured by a spectrophotometer at a wavelength of 280 nm.

### 2.7. RT-PCR Studies

The relative mRNA expressions of samples were determined by the Comparative CT method of qPCR using the TakyonTM One-Step Rox Probe 5X MasterMix dTTP kit (Eurogentec, Seraing, Belgium) with primers *Abcb1a* (Rn01639253_m1), *Abcb1b* (Rn01529252_g1), *Abcg2* (Rn00710585_m1), *Cyp19a1* (Rn00567222_m1), and *Ar* (Rn00560747_m1), respectively. As an endogenous control, *β-actin* (Rn00667869_m1) was used. Plate reactions were performed by ABI StepOne Real-Time cycler (Thermofisher, Hungary), starting with one cycle at 48 °C for 10 min (reverse transcription) and one cycle at 95 °C for 3 min (Taq polymerase activation) then 55 cycles at 95 °C for 10 s (denaturation) and 60 °C for 1 min (annealing/elongation).

### 2.8. Western Blot Analysis

Protein samples of 50 µg per well was electrophoresed and blotted on nitrocellulose membranes then antibody binding was detected with the WesternBreeze Chromogenic Western blot immunodetection kit (ThermoFisher Scientific, Hungary) as described earlier [16]. Antibodies with the appropriate dilution were used for the detection of P-gp (34 kDa, 1:200, abx177960, Abbexa Ltd., Cambridge, UK), BCRP (72 kDa, 1:500, SAB5701106, Merck Life Science Ltd., Budapest, Hungary), and β-actin (42 kDa, 1:1000, bs-0061R, Bioss Antibody, Beijing, China).

### 2.9. ELISA Assay

Testicular testosterone levels were measured using the Rat T (Testosterone) ELISA kit (ER1462, FineTest, Wuhan, China) as described in the assay guide, and, according to the manufacturer’s recommendation, 150 µg of protein was diluted and added to each well. The intensity of the samples was determined with SPECTROStar Nano microplate spectrophotometer (BMG Labtech, Ortenberg, Germany) at a wavelength of 450 nm, and sample concentrations were calculated by curve fitting using Prism 9.0 software (Graphpad Software Inc., San Diego, CA, USA).

### 2.10. Statistical Evaluation

Statistical analysis was performed using Prism 9.0. Data were presented as mean + standard error of the mean (SEM) and analyzed using an unpaired *t*-test or two-way ANOVA followed by Bonferroni correction. Statistical significance was approved at *p* < 0.05.

## 3. Results

### 3.1. The Characteristic of the Diet-Induced Obese Rat Model

**Figure 2:**
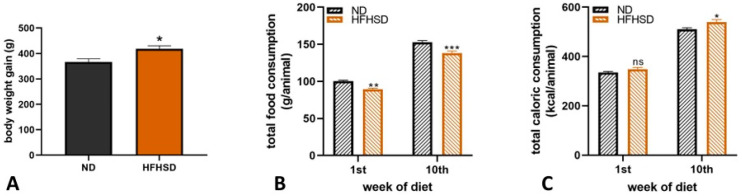
Changes in body weight gain (**A**), food (**B**), and caloric (**C**) consumption during the nutritional intervention between the normal diet (ND) and high-fat, high-sugar diet (HFHSD) group. Data are presented as means ± SEM and statistical significance was accepted at *p* < 0.05 compared to the ND group (ns *p* > 0.05, * *p* < 0.05, ** *p* < 0.01, *** *p* < 0.001).

During the 10-week nutritional intervention period, significant weight gain (Figure 2A) was observed in the HFHSD group compared to the ND group. The total food consumption was significantly lower in the HFHSD group than in the controls at the beginning of the diet and at the end of the experiment (Figure 2B). However, total caloric intake was higher in the HFHSD group (Figure 2C).

**Figure 3:**
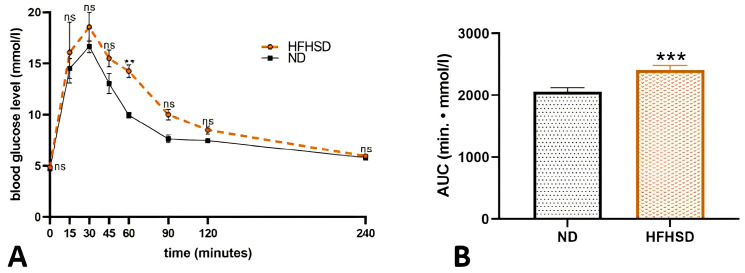
Blood glucose levels (**A**) and area under curve (AUC) analysis (**B**) of normal diet (ND) and high-fat, high-sugar diet (HFHSD) male rats at 9 weeks of age. Data are presented as means ± SEM and statistical significance was accepted at *p* < 0.05 compared to the ND group (ns *p* > 0.05, ** *p* < 0.01, *** *p* < 0.001).

A glucose tolerance test was performed at 9 weeks of age, showing higher blood glucose levels at all time points, with a significant elevation at 60 min in obesity (Figure 3A). Furthermore, the area under the curve (AUC) was significantly increased in the HFHSD-fed animals (Figure 3B), indicating worse glucose tolerance.

**Figure 4:**
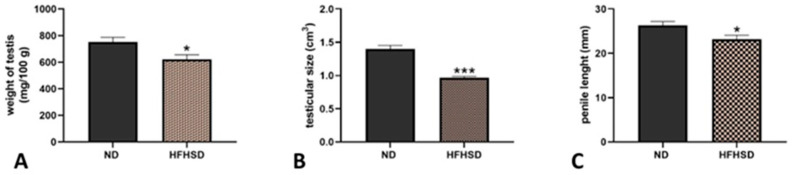
Changes in testicular weight (**A**), testicular size (**B**) and penile length (**C**) in normal diet (ND) and high-fat, high-sugar diet (HFHSD) animals. Data are presented as means ± SEM and statistical significance was accepted at *p* < 0.05 compared to the ND group (* *p* < 0.05, *** *p* < 0.001).

At the end of the dietary intervention, we observed a significant reduction in testicular weight (Figure 4A), testicular size (Figure 4B), and penile length (Figure 4C) in the HFHSD group compared with controls.

### 3.2. Effect of HFHSD on Testicular ABC Transporters

**Figure 5:**
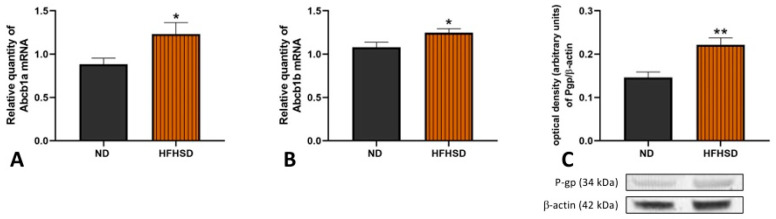
Changes in *Abcb1a* (**A**), *Abcb1b* (**B**) mRNA and P-gp (**C**) protein expression in the testicles of normal diet (ND) and high-fat, high-sugar diet (HFHSD) animals. Data are presented as means ± SEM and statistical significance was accepted at *p* < 0.05 compared to the ND group (* *p* < 0.05, ** *p* < 0.01).

Testicular *Abcb1a* (Figure 5A) and *Abcb1b* (Figure 5B) mRNA and P-gp protein (Figure 5C) expressions were significantly increased in the HFHSD animals compared to controls. The original pictures of the nitrocellulose membranes of Western blotting are attached in the Supplementary Materials (Supplementary Figure S1).

Based on PCR cycle threshold (C_T_) values, the *Abcb1b* isoform has significantly higher C_T_ values in rat testicular tissues than the *Abcb1a* isoform across all experimental groups (Table 1). Higher C_T_ values represent less detected mRNA, while more cycles of amplification are needed to detect the target. Based on the C_T_ values, *Abcb1a* is presented in higher quantities than *Abcb1b* in the testes of both ND and HFHSD rats, suggesting the dominance of *Abcb1a* mRNA isoform.

**Figure 6:**
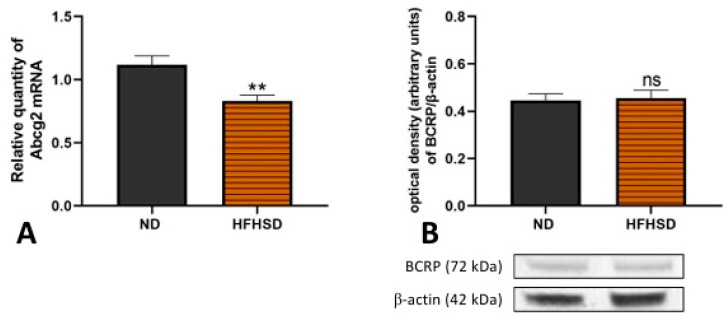
Changes in *Abcg2* (**A**) mRNA and BCRP (**B**) protein expression in testicles of normal diet (ND) and high-fat, high-sugar diet (HFHSD) animals. Data are presented as means ± SEM and statistical significance was accepted at *p* < 0.05 compared to the ND group (ns *p* > 0.05, ** *p* < 0.01).

The mRNA expression of testicular *Abcg2* (Figure 6A) was significantly decreased, while BCRP protein expression (Figure 6B) remained unchanged in the obese animals. The original pictures of the nitrocellulose membranes of Western blotting are attached in the Supplementary Materials (Supplementary Figure S1).

### 3.3. Effect of HFHSD on Spermatic ABC Transporters

**Figure 7:**
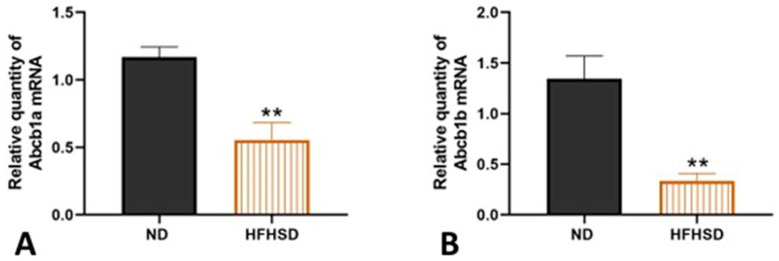
Changes in *Abcb1a* (**A**) and *Abcb1b* (**B**) mRNA expression in sperms of normal diet (ND) and high-fat, high-sugar diet (HFHSD) animals. Data are presented as means ± SEM and statistical significance was accepted at *p* < 0.05 compared to the ND group (** *p* < 0.01).

The spermatic *Abcb1a* (Figure 7A) and *Abcb1b* (Figure 7B) mRNA expressions significantly decreased in the HFHSD animals compared to the controls.

Detection of *Abcb1a* and *Abcb1b* isoforms in sperm required more amplification cycles, indicating lower levels than in the testis (Supplementary Table S1). The C_T_ values of *Abcb1a* and *Abcb1b* were similar in both experimental groups (Table 2).

**Figure 8:**
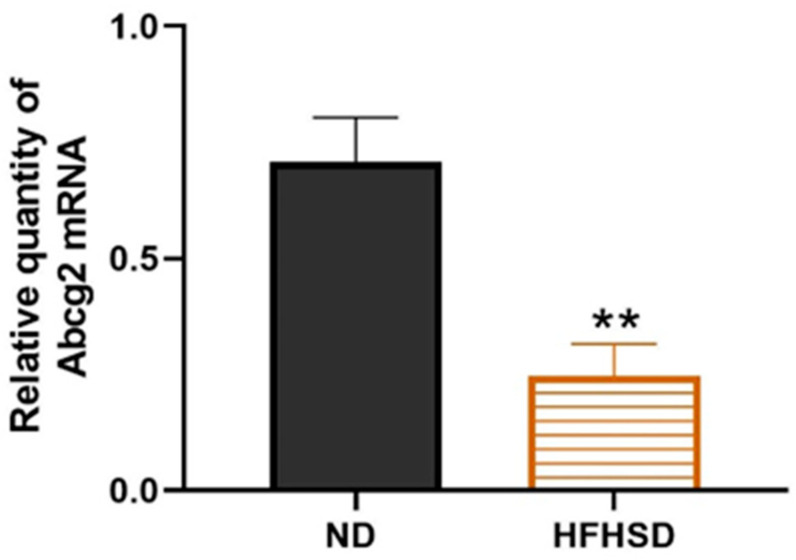
Changes in *Abcg2* mRNA expression in sperms of normal diet (ND) and high-fat, high-sugar diet (HFHSD) animals. Data are presented as means ± SEM and statistical significance was accepted at *p* < 0.05 compared to the ND group. (** *p* < 0.01).

The spermatic *Abcg2* mRNA expression was reduced by a third in the HFHSD animals compared to the controls (Figure 8). According to the C_T_ values, a lower amount of *Abcg2* was detected in the sperm than in the ND group (Supplementary Table S1).

### 3.4. Effect of HFHSD on Testicular Androgen State

**Figure 9:**
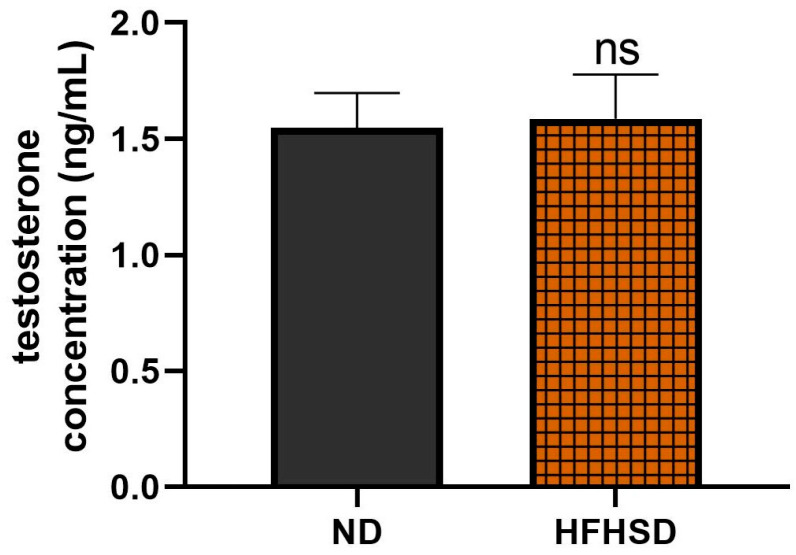
Changes in testosterone concentration in testicles of normal diet (ND) and high-fat, high-sugar diet (HFHSD) animals. Data are presented as means ± SEM and statistical significance was accepted at *p* < 0.05 compared to the ND group (ns *p* > 0.05).

The testicular testosterone concentration remained unchanged in rats fed by HFHSD at the end of the experiment compared to the normal diet groups (Figure 9).

**Figure 10:**
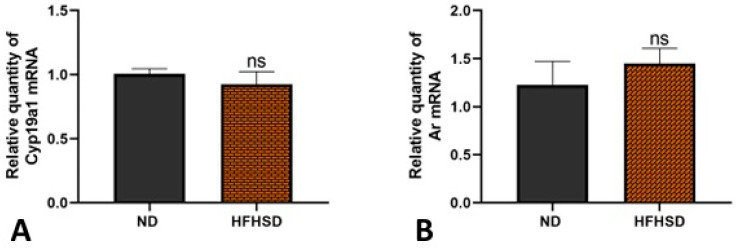
Changes in *Cyp19a1* (**A**) and *Ar* (**B**) mRNA expressions in testicles of normal diet (ND) and high-fat, high-sugar diet (HFHSD) animals. Data are presented as means ± SEM and statistical significance was accepted at *p* < 0.05 compared to the ND group (ns *p* > 0.05).

In the HFHSD animals, neither testicular *Cyp19a1* (Figure 10A) nor *Ar* (Figure 10B) mRNA expressions changed significantly compared to the ND animals after the 10-week dietary intervention.

## 4. Discussion

In parallel with the increased prevalence of obesity, the incidence of infertility also increased globally in the last few decades, indicating their interconnected relationship [42]. Feeding a high-fat, high-sugar diet to rodents is a widely accepted and reliable dietary method to induce human-like obesity with metabolic disturbances [43,44]. Our HFHSD-fed animals showed higher weight gain with increased caloric consumption and worse glucose tolerance during the experiment, which confirmed the presence of obesity and the prediabetic state. These results are in accordance with the previous studies, highlighting the validity of the obese rat model [16,37,38,45].

The 10-week dietary intervention spanned the rats’ sexual maturation period, and the obesogenic diet reduced the relative weight of the testes. Although a previous study reported unaltered testicular weight in rats fed a high-fat diet for 15, 30, or 45 weeks, more studies with shorter dietary periods revealed similar testicular weight reduction as we observed. The differences between these studies are presumably the result of differences in diet composition [46,47,48]. The HFHSD males also showed smaller testicular size and penile length, suggesting structural impairments in these genitals. These results are in accordance with the human studies that those children and adolescents who are obese during puberty are associated with reduced penile and testicular development [49,50]. Our findings further strengthen the human translational potential of the model and highlight the disruptive effects of obesity on genital development during the pubertal period.

P-gp and BCRP efflux transporters are pivotal proteins in the testicular tissue, especially in the Sertoli cells. Their primary role is to maintain the physiological milieu for sperm development in the seminiferous tubules, and their function is strongly correlated with BTB integrity [51]. In our studies, we revealed increased testicular *Abcb1a* and *Abcb1b* mRNA and P-gp protein expression in the obese rat testicular tissues with an *Abcb1a* isoform dominance in both diet groups, similar to the reported literature data [52]. In contrast, mRNA expression of testicular *Abcg2* decreased, while the protein level remained unchanged. A previous study reported upregulated P-gp expression in human Sertoli cells from oligoasthenozoospermic males, who exhibit decreased spermatozoa counts, reduced motility, and altered morphology [27]. Although we did not investigate detailed sperm parameters, previous studies have established that diet-induced obese rat models also show reduced fertility rates with altered sperm parameters, as in humans [53,54,55]. According to earlier studies, *Abcb1a*, *Abcb1b*, and *Abcg2* are expressed in the rat spermatozoa [28,29], and we investigated the mRNA expression of these spermatic ABC transporters to evaluate if there were any changes that indicate reduced fertility. We revealed a dramatic reduction in both *Abcb1a*, *Abcb1b*, and *Abcg2* levels and also reported that these transcripts are present in lower quantities in the sperm than in the testis. The reduced expressions of the investigated spermatic transporters suggest the decline of fertilizing ability of the sperm, since both transporters are involved in the regulation of sperm protection, motility, capacitation, or acrosome reaction [28,29,30,31]. Presumably, the lower spermatic *Abcg2* expression in HFHSD rats may reduce cholesterol efflux, which may contribute to the higher cholesterol content, reduced motility, and impaired acrosome reaction observed in sperm from obese patients [56]. Since P-gp also transports endogenous sterols [29], *Abcb1a* and *Abcb1b* dysregulations may also interfere with sperm capacitation and acrosome reaction in obesity.

The association between testicular P-gp upregulation and the changes related to reduced fertility ability of sperm may be explained by mechanisms beyond xenobiotic efflux. P-gp also transports endogenous substrates, such as androgens, including testosterone [17,18], which are essential in spermatogenesis [57]. A more pronounced increase in P-gp expression could restrict testosterone penetration across the BTB, leading to a relative deficiency in the seminiferous tubules and reduced fertility performance. This hypothesis is further supported by the changes in *Abcg2* expression. Since *Abcg2* can be regulated by testosterone [36], decreased *Abcg2* mRNA transcription may reflect reduced testosterone penetration into the Sertoli cell.

To strengthen this hypothesis, we also measured the testicular androgen status by the determination of testosterone concentration, *Cyp19a1*, and *Ar* mRNA levels, as obesity in males is associated with decreased serum testosterone levels in a BMI-dependent manner, primarily due to reduced testosterone production in the testes or increased conversion of peripheral testosterone to estrogen by the aromatase enzyme, primarily in adipose tissue [10]. We revealed that neither altered testosterone secretion nor conversion, nor androgen responsiveness, is disrupted in testicular tissues at the end of the obesogenic diet, which confirms our theory. In diet-induced obese rodents, serum testosterone typically decreases; however, the rate of change depends on the duration of diet feeding, the extent of weight gain, and the composition of the obesogenic diet [53,55,58,59,60,61]. Presumably, the 10-week dietary interval was insufficiently long to develop severe obesity, which may explain the unchanged testicular androgen state. At the same time, our data suggest that mild–moderate obesity caused by short-term obesogenic diet has already induced infertility-related changes in reproductive organs and sperm development, indicating predisposition to fertility impairments despite the fact that testicular androgen dysregulation has not occurred yet.

It is also important to mention that several studies revealed that absence [23,24,25,62,63,64,65,66], polymorphism [19,32], or decreased activity [26,67] of these testicular transporters leads to higher penetration of their potentially harmful substrates and could induce reproductive toxicity, which is also associated with male infertility [68]. As these transporters regulate both the entry and the exit of various substances in the testicular tissue [14], too low and too high expression or activity can both cause fertility problems by letting in too many harmful substances and damaging the proper sperm development, or keeping out essential substances, resulting in a deficiency of vital elements and disrupting the spermatogenesis in the seminiferous tubules. On the other hand, testicular P-gp upregulation could also be a problem in pharmacotherapy, particularly in antiretroviral treatment. Several antiretroviral drugs are P-gp substrates, like abacavir or zidovudine. Since testicular tissue is an ideal anatomic reservoir for human immunodeficiency virus due to the BTB [69,70], increased P-gp expression could limit the distribution of antiretrovirals into testicular tissue, leading to suboptimal drug exposure and viral persistence in obesity. Based on these, targeting P-gp with specific modulators could be promising in the treatment or prevention of obesity-related male infertility and enhance drug penetration across the blood–testis barrier in obesity. Although testicular BCRP protein expression did not change in the obese rats, in contrast to the mRNA expression, the permanent reduction in transcription could manifest in protein level changes with the progression of obesity, which should be explored with longer dietary intervention.

### Limitations

Our findings are subject to limitations that warrant consideration. The study lacks transport-function examinations, limiting the precise interpretation of the data obtained from ABC transporters. In vivo pharmacokinetic and pharmacodynamic studies with specific substrates and inhibitors would be needed to evaluate the testosterone concentration in the seminiferous ducts. Detailed sperm analysis would also be required, including sperm count, motility, morphology, and protein expression profile, to extend the model’s fertility characteristics. Finally, a comparison of these results with longer dietary intervention periods would also be beneficial for assessing the impact of obesity on these factors.

## 5. Conclusions

**Figure 11:**
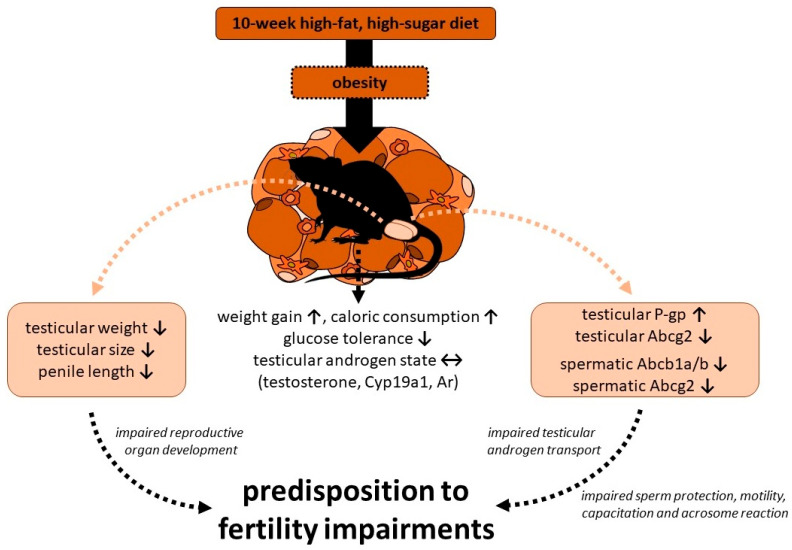
Obesity induced by short-term diet triggered infertility-related changes without androgen dysregulation. ↓: decrease; ↑: increase; ↔: no change.

Our finding show that a 10-week high-fat, high-sugar diet induced mild–moderate obesity with metabolic disturbances in male rats triggers impaired reproductive organ size and infertility-related changes in testicular and spermatic ABC transporters without androgen dysregulation of testes (Figure 11).

We believe that these obesity-induced changes may predispose men to fertility impairments even before significant hormonal changes occur. Future investigation on these factors could be promising in the treatment or prevention of obesity-related male infertility and the optimization of drug therapy in obesity.

## Figures and Tables

**Table 1:** Differences in cycle threshold (C_T_) values of *Abcb1a* and *Abcb1b* in testes from normal diet (ND) and high-fat, high-sugar diet (HFHSD)-fed rats. Values are number of mean ± standard deviation (SD). *** *p* < 0.001 compared to the *Abcb1a* isoform in each experimental group.

Diet Group	Average C_T_ Value of *Abcb1a*	Average C_T_ Value *Abcb1b*
ND	23.803 ± 0.240	27.055 ± 0.238 ***
HFHSD	23.548 ± 0.532	26.607 ± 0.846 ***

**Table 2:** Differences in cycle threshold (C_T_) values of *Abcb1a* and *Abcb1b* in sperm from normal diet (ND) and high-fat, high-sugar diet (HFHSD)-fed rats. Values are number of mean ± standard deviation (SD). ns *p* > 0.05 compared to the *Abcb1a* isoform in each experimental group.

Diet Group	Average C_T_ Value of *Abcb1a*	Average C_T_ Value *Abcb1b*
ND	40.245 ± 0.738	39.553 ± 1.468 ^ns^
HFHSD	39.769 ± 0.225	39.246 ± 1.439 ^ns^

## Data Availability

All data accessed and analyzed in this study are available in the article and its Supplementary Materials. Further information and requests for resources and reagents should be directed to and will be fulfilled by the lead contact, Eszter Ducza (ducza.eszter@szte.hu).

## Supplementary Materials

The following supporting information can be downloaded at: https://www.mdpi.com/article/10.3390/nu18111829/s1, Figure S1: Representative unedited gel photos of testicular P-glycoprotein (P-gp) and breast-cancer resistance protein (BCRP) expression in normal diet (ND) and high-fat-high-sugar diet (HF) fed rats; Table S1: Differences in cycle threshold (C_T_) values of *Abcb1a Abcb1b* and *Abcg2* in testes and sperm from normal diet (ND) and high-fat, high-sugar diet (HFHSD) fed rats.

## Abbreviations

The following abbreviations are used in this manuscript:

Ar	androgen receptor
ABC	ATP-binding cassette
AUC	area under the curve
BTB	blood–testis barrier
BCRP	breast cancer resistance protein
Cyp19a1	cytochrome P450-dependent aromatase enzyme
C_T_	cycle threshold
GTT	glucose tolerance test
HFHSD	high-fat, high-sugar diet
ND	normal diet
P-gp	P-glycoprotein
SD	standard deviation
SEM	standard error of the mean
