# Supplementary material for: Obesity Triggers Dysregulation of Essential ABC Transporters in Rat Testis and Sperm
Source: Nutrients. 2026 Jun 5;18(11):1829. doi: 10.3390/nu18111829 (PMC13258864; doi:10.3390/nu18111829)
Supplement: Supplementary file 1 [file nutrients-18-01829-s001.zip › nutrients-4330919-supplementary.pdf]

Obesity Triggers Dysregulation of Essential ABC Transporters in Rat Testis and Sperm

Péter Szatmári[1], Kata Kira Kemény[1], Adrienn Seres-Bokor[1] and Eszter Ducza[1,*]

[1]Department of Pharmacodynamics and Biopharmacy, Faculty of Pharmacy, University of Szeged, 6720 Szeged, Hungary, Eötvös street 6.; P. Sz. (szapeti40@gmail.com), K. K. K. (kemeny.kata.kira@szte.hu), A. SB. (seres-bokor.adrienn@szte.hu), E. D. (ducza.eszter@szte.hu)

* Corresponding author: ducza.eszter@szte.hu

Supplementary Figure S1: Representative unedited gel photos of testicular P-glycoprotein (P-gp) and breast-cancer resistance protein (BCRP) expression in normal diet (ND) and high-fat-high-sugar diet (HF) fed rats. *PageRuler™ Prestained Protein Ladder (Thermo Fisher Scientific, Hungary) was used as a size standard. After gel electrophoresis, proteins were transferred to a nitrocellulose membrane, then dyed with Ponceau S Staining Solution. Membranes were cut into strips at the appropriate molecular weights, and proteins were detected by antibody binding under the same conditions. The representative gel photos originate from the same Western blot gel.*

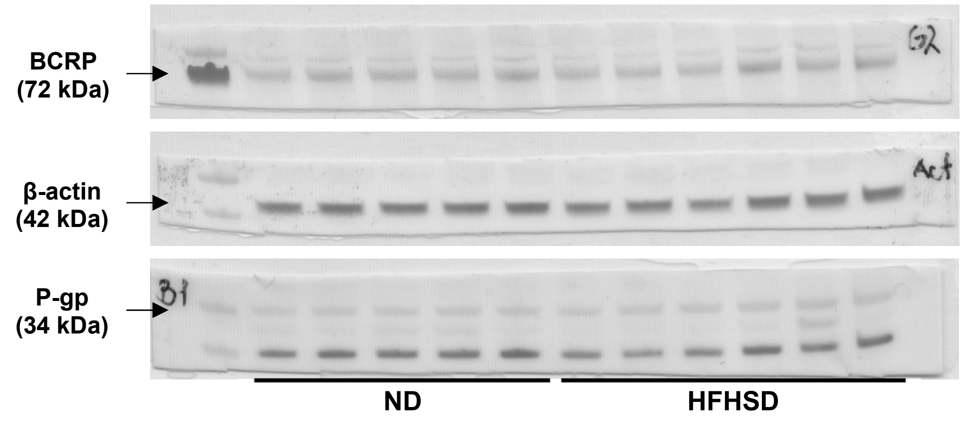

Supplementary Table S1. Differences in cycle threshold (C_T) values of Abcb1a Abcb1b and Abcg2 in testes and sperm from normal diet (ND) and high-fat, high-sugar diet (HFHSD) fed rats. *Values are number of mean ± SD. *** p<0.001 compared to the testicular isoform in each experimental group.*

Isoform	ND		HFHSD	
	testis	sperm	testis	sperm
**Abcb1a (C_T value)**	23.803 ± 0.240	40.245 ± 0.738 ***	23.548 ± 0.532	39.553 ± 1.468 ***
**Abcb1b (C_T value)**	27.055 ± 0.238	39.769 ± 0.225 ***	26.607 ± 0.846	39.246 ± 1.439 ***
**Abcg2 (C_T value)**	21.783 ± 0.144	35.039 ± 0.968 ***	21.539 ± 0.170	34.517 ± 0.773 ***
